# A first linkage map and downy mildew resistance QTL discovery for sweet basil (*Ocimum basilicum*) facilitated by double digestion restriction site associated DNA sequencing (ddRADseq)

**DOI:** 10.1371/journal.pone.0184319

**Published:** 2017-09-18

**Authors:** Robert Pyne, Josh Honig, Jennifer Vaiciunas, Adolfina Koroch, Christian Wyenandt, Stacy Bonos, James Simon

**Affiliations:** 1 Department of Plant Biology, Rutgers University, New Brunswick, New Jersey, United States of America; 2 Science Dept., Borough of Manhattan Community College, The City University of New York, New York, NY, United States of America; Nanjing Forestry University, CHINA

## Abstract

Limited understanding of sweet basil (*Ocimum basilicum* L.) genetics and genome structure has reduced efficiency of breeding strategies. This is evidenced by the rapid, worldwide dissemination of basil downy mildew (*Peronospora belbahrii*) in the absence of resistant cultivars. In an effort to improve available genetic resources, expressed sequence tag simple sequence repeat (EST-SSR) and single nucleotide polymorphism (SNP) markers were developed and used to genotype the MRI x SB22 F_2_ mapping population, which segregates for response to downy mildew. SNP markers were generated from genomic sequences derived from double digestion restriction site associated DNA sequencing (ddRADseq). Disomic segregation was observed in both SNP and EST-SSR markers providing evidence of an *O*. *basilicum* allotetraploid genome structure and allowing for subsequent analysis of the mapping population as a diploid intercross. A dense linkage map was constructed using 42 EST-SSR and 1,847 SNP markers spanning 3,030.9 cM. Multiple quantitative trait loci (QTL) model (MQM) analysis identified three QTL that explained 37–55% of phenotypic variance associated with downy mildew response across three environments. A single major QTL, *dm11*.*1* explained 21–28% of phenotypic variance and demonstrated dominant gene action. Two minor QTL *dm9*.*1* and *dm14*.*1* explained 5–16% and 4–18% of phenotypic variance, respectively. Evidence is provided for an additive effect between the two minor QTL and the major QTL *dm11*.*1* increasing downy mildew susceptibility. Results indicate that ddRADseq-facilitated SNP and SSR marker genotyping is an effective approach for mapping the sweet basil genome.

## Introduction

Sweet basil (*Ocimum basilicum* L.) is the most widely cultivated and economically salient *Ocimum* species in the United States and Europe [1]. Annual US revenue generated from sweet basil and other culinary herbs sold as bedding plants are estimated to be 96.8 million dollars [2]. Introduction of basil downy mildew (*Peronospora belbahrii*) to Europe in 2001 [3] and the United States in 2007 [4] has resulted in wide spread crop destruction and an estimated tens of millions of dollars in economic losses in the US alone [5]. Absence of effective seed treatment or chemical control measures has aided rapid dissemination of this pathogen to major production areas worldwide [6–13]. Lack of economically sustainable conventional, organic or cultural control, potential for fungicide resistant pathogen evolution [7,14] and consistent disease presence in major growing regions create a compelling rationale for the development of genetic resistance to downy mildew in sweet basil.

Multiple publications have identified downy mildew resistance within the *Ocimum* genus [15,16] and Ben-Naim et al. [17] demonstrated introgression into *O*. *x basilicum* F1 hybrids. Although possible, hybridization of commercial *O*. *basilicum* varieties with resistant genotypes is largely met with F1 sterility or sexual incompatibility [17,18]. Despite these challenges, an initial characterization of downy mildew resistance was provided by a multi-site field trial that evaluated F_2_ and backcross generations derived from a cross between downy mildew resistant inbred cultivar Mrihani (MRI) and susceptible inbred Rutgers University breeding line SB22 [19]. These efforts generated important results that have helped to inform an effective breeding program targeting genetic resistance. Nevertheless, a resistant commercial sweet basil variety remains to be seen 15 years after the first report of *P*. *belbahrii*, reflecting the difficulties currently associated with genetic improvement of this poorly characterized plant species.

Linkage map construction and subsequent association of DNA markers linked to important traits, or QTL, are becoming essential components of modern plant breeding programs. Although increasingly common in other horticultural species, neither a linkage map nor QTL have been developed for sweet basil. Lack of insight into the rather large [20] and potentially complex *O*. *basilicum* genome render sweet basil an unattractive species for genetic studies. Fortunately the rapid rise and plummeting costs of next-generation sequencing (NGS) have made high-throughput single nucleotide polymorphism (SNP) discovery accessible to non-model species [21,22]. Moreover, the introduction of reduced representation NGS strategies through restriction site associated DNA sequencing (RADseq) has revolutionized population-level genetic studies by providing a uniformly distributed subset of the genome across pooled individuals [23,24]. The combination of RADseq techniques with increasingly robust data analysis software [22,25,26] has provided unprecedented access to the genomes of complex species for linkage mapping. This includes polyploid species, which can generally be divided into autopolyploid (whole genome duplication following intraspecific hybridization) or allopolyploid (whole genome duplication following interspecific hybridization) classes. Allopolyploids are more easily targeted for linkage mapping because the segregation of loci usually occurs within divergent sub-genomes, which allows for separation of homologous from homeologous loci [27,28]. By dividing segregating loci among sub-genomes one can obtain a dataset compatible with traditional diploid map construction software packages (Joinmap, RQTL, etc.). Genotyping allopolyploids is typically performed with co-dominant markers such as simple sequence repeats (SSRs) or single nucleotide polymorphisms (SNPs) by isolation of single-locus (single sub-genome) markers [29] or development of a model for allele sub-genome assignment [30,31].

*Ocimum basilicum* is an outcrossing [32] tetraploid [18,33] that has demonstrated disomic inheritance for multiple traits [34,35], suggesting a diploidized polyploid genome. This allopolyploid hypothesis is supported by cytological evidence that demonstrated preferential pairing of *O*. *basilicum*, *O*. *americanum* (syn. *O*. *canum*) and their F_1_ hybrid [18]. Furthermore, an initial investigation indicated that basil downy mildew resistance conferred genotype MRI segregates in disomic fashion [19]. Major gene control is a common form of host resistance among plant species and has been demonstrated through QTL discovery in lettuce [36–38], spinach [39], melon [40], and grape [41].

In this study, a set of genic SSR markers were developed from the currently available National Center for Biotechnology Information (NCBI) EST database and used to genotype a sweet basil F_2_ mapping population from a cross between MRI and downy mildew susceptible genotype SB22 following an allotetraploid segregation model. A double digestion RADseq (ddRADseq) approach was then employed for SNP discovery and *de novo* genotyping. Genotype data was subjected to a filtration process to retain bi-allelic, homologous polymorphic loci to generate an intercross diploid dataset. Resulting genotype data were used to construct the first linkage map for sweet basil, which is anchored by SSRs and saturated by SNPs. Multiple QTL analyses were performed to identify genomic regions with association to downy mildew (*P*. *belbahrii*) resistance.

## Materials and methods

### Plant material

An F2 mapping population was developed in 2014 from a cross between inbred genotypes MRI (♀) and SB22 (♂) as previously described [19]. SB22 is an inbred line selected for tolerance to Fusarium wilt (*Fusarium oxysporum f*. *sp*. *basilica*) and is highly susceptible to *P*. *belbahrii*, while MRI is an inbred line resistant to *P*. *belbahrii*. The F_1_ hybrid and 104 F_2_ individuals were randomly selected and maintained as vegetative cuttings in Rutgers University research greenhouses (New Brunswick, NJ, U.S.A). This allowed for clones of each individual to be field transplanted for phenotyping across multiple years and locations.

### Genotyping

Genomic DNA (gDNA) was extracted from the grandparents, F_1_ and 104 F_2_ individuals using ~80 mg of young ground leaf tissue using the E.N.Z.A. SP Plant DNA Kit (Omega BioTek, Norcross, GA). DNA was quantified and assessed for quality by measurement of 260/280 and 260/230 absorbance ratios using a Nanodrop (Thermo Scientific, Waltham, MA).

#### EST-SSR analysis

The National Center for Biotechnology Information (NCBI) *O*. *basilicum* expressed sequence tag (EST) database of 23,845 cDNA sequences was downloaded and assembled using CAP3 software [42] with default settings. The resulting contig and remaining singlet sequences were mined with SciRoKo software [43] for di-, tri- and tetranucleotide repeat sequences with a minimum of 10 nucleotides. SSRs meeting this criteria were selected for the presence of ≤300 bp flanking sequences that were subsequently used for primer pair design with Primer3 software [44]. This pipeline produced 811 putative SSR markers from which a subset of 89 di-, 115 tri-, and 36 tetranucleotide were used in this study. Primer pairs were synthesized (Integrated DNA Technologies, Coralville, IA) with the 5’ end of forward primers appended with the M13 (-21) sequence (5’-TGTAAAACGACGGCCAGT-3’)[45] to facilitate fluorescent labeling of PCR products. The 5’ end of reverse primers were “pig-tailed” with the 5’-GTTTCTT-3’ sequence [46] to ensure consistent polyadenylation across reactions.

PCR amplification for all reactions included 5 ng of gDNA, 10x Ramp-Taq PCR buffer (Denville Scientific, Metuchen, NJ), 2.0 mM MgCl2, 0.25 mM each dNTP (Denville Scientific), 0.5 U Ramp-Taq DNA polymerase (Denville Scientific), 0.5 pmol forward primer, 1.0 pmol reverse primer, and 1.0 pmol fluorescently labeled (FAM, NED, PET, or VIC) M13(-21) primer. Template gDNA was amplified using the following conditions: initial denaturation of 94°C for 5 min, followed by 30 cycles of 94°C for 30 sec, 55°C for 45 sec, 72°C for 45 sec, followed by 20 cycles of 94°C for 30 sec, 53°C for 45 sec, 72°C for 45 sec, followed by a final extension of 72°C for 10 min. GeneScan 600 LIZ (Applied Biosystems) size standard was added to resulting PCR products and separated by capillary electrophoresis on an ABI 3500xL Genetic Analyzer (Life Technologies Corporation, Carlsbad, CA). PCR product fragment length measurement and allele binning and was performed using Genemapper 4.1 software (Applied Biosystems).

PCR was performed in duplicate for 240 SSR markers initially using only MRI and SB22 grandparent gDNA to select for markers resulting in unambiguous PCR products (e.g., absent of any non-specific binding) and polymorphism. Markers fulfilling these criteria were subsequently used to evaluate the F_2_ mapping population. SSR markers having ≥3 amplicons for either grandparent were discarded and the remaining SSRs (containing either one or two amplicons per grandparent) were tested for chi-square goodness of fit to 1:2:1 or 3:1 diploid segregation models.

#### SNP analysis

An initial experiment was performed to compare two and three enzyme library preparation approaches using gDNA from 22 F_2_ individuals. The double digestion RADseq libraries were prepared according to Poland et al. [47] using rare-cutting *PstI* (NEB, USA) and the more common-cutting *MspI* (NEB, USA). In the three-enzyme digestion, the Poland et al. [47] protocol was modified to include *ApeKI*, which serves as a cutter to further reduce the complexity of the genome. Due to the addition of this enzyme, a 2 hour, 75°C incubation followed the initial digestion of gDNA with *PstI* and *MspI*. For both the two- and three-enzyme approaches, the *PstI*-complementary forward adapter and *MspI*-complementary reverse Y-adapters were added to the ligation reaction to ensure that only *PstI-MspI* fragments would be amplified during PCR, while all other digested fragments (*MspI-MspI*, *MspI*-*ApeKI*, *PstI-ApeKI* and *PstI- PstI*) should fail to amplify. Both prior to PCR and following library pooling, samples were mixed with 0.5 v/v Agencourt AMPure XP Beads (Beckman Coulter, USA) and washed with 70% ethanol to remove fragments sized less than 300 bp. All *PstI* and *MspI* adaptors included previously published barcodes [47] to uniquely identify individual samples.

All final libraries were prepared using the double digestion method rather than the triple digestion method, which generated library concentrations too low for sequencing. Four separate libraries were prepared for each grandparent to generate a 4x sequencing depth relative to the F1 and F2 progeny. Libraries for MRI (4x), SB22 (4x), F1 and 100 F2 individuals were quantified using Qubit (Life technologies, Grand island, NY). All samples were normalized to 5 ng/uL before pooling. This pool (109-plex) was paired-end sequenced on two Hi-Seq 2000 (Illumina, USA) lanes, once in Rapid-Run mode (2x150bp) and once in High-Output mode (2x100).

The Stacks (v1.3) software [48] pipeline was used to convert raw reads into genotype data. Concatenated single and paired end read fastq files were trimmed to 75 bp, quality filtered with the ‘-q’ flag and de-multiplexed using the default settings of the Stacks process_radtags.pl program. The ustacks program was then used to assemble matching reads across all samples and call SNPs within each group of reads (Stack) to generate individual haplotype alleles. A minimum of 3 matching reads (-m) was required to create a Stack with a maximum nucleotide mismatch allowance (-M) of 3 for F_2_ and grandparent stacks. The cstacks program generated a Catalog from Stacks that were polymorphic among the grandparents (MRI and SB22) to which F_2_ progeny haplotypes were matched using the sstacks program to identify putative loci. Loci missing >20 genotyped progeny were excluded from subsequent analyses.

A potential pitfall in polyploid SNP genotyping is the vulnerability of paralogous sequence variants being called as false positive polymorphic loci [27]. In an approach similar to Hohenlohe et al. [28], the Stacks web user interface was used to exclude loci with >1 SNP and significant (p≤0.10) deviation from an 1:2:1 ratio expected for an F_2_ intercross between dual-homozygous grandparents. This approach was therefore employed to filter paralogs and obtain a bi-allelic SNP dataset for genetic mapping.

### Linkage map construction

Linkage map construction was performed using Joinmap 4.1® (Kyazma, NL) [49] with genotype data coded as an F_2_ intercross population type. Grouping was performed using independence logarithm of odds (LOD) scores from 2 to 15 with a step of 1, after which a minimum LOD score of 10 was used to determine autonomous linkage groups (LGs). Placement of loci was determined by comparison of map orders derived from the multipoint maximum likelihood and regression algorithms in Joinmap 4.1® (Kyazma, NL). An initial map was constructed using the maximum likelihood mapping function with parameters adjusted from the default settings when necessary to allow the algorithm to converge [50]. A second map was generated by regression mapping using a minimum LOD score of 4.0, recombination frequency of 0.35 and ripple jump threshold of 5.0. Maximum likelihood and regression maps were compared to identify suspect loci that might be misplaced. A locus or group of loci demonstrating major differences in map order location were removed to provide robust support for loci placement in the final map, which was estimated by maximum likelihood method.

### Phenotyping

Downy mildew response for all individuals in this study was measured over two years and at two field locations by assessing the severity of abaxial leaf sporulation as described by Pyne et al. [19]. Field phenotyping experiment locations were selected based consistent annual disease pressure [19,51] and susceptible check plants. Susceptible control cultivar ‘DiGenova’ was included in the experimental design and overhead irrigation was applied as needed to provide uniform disease severity. Six leaves per individual were randomly sampled and scored using an ordered categorical scale of 0–4 (0 = no sporulation, 1 = 1–10%, 2 = 11–25%, 3 = 26–50%, 4 = 51–100%). Individuals were assigned a value between 0 (lowest possible severity) and 1 (highest possible severity) by dividing by a maximum score of 24. Data were collected in 2014 at Northern (NJSN14) and Southern (NJRA14) New Jersey locations [19] and the Southern New Jersey location in 2015 (NJRA15). All experiments were performed in randomized complete block design with three replications. Each of the 94 genotyped F_2_ individuals were phenotyped in NJRA14 and NJSN14, while 80 were phenotyped in NJRA15 due to plant death during the one year period between experiments. Phenotypic data from a single date corresponding to the highest F_2_ population mean disease severity were selected for each unique year x location combination (environment) for subsequent QTL analyses. Phenotype data was square root transformed and individuals not included in 2015 phenotyping were scored as missing for QTL analysis.

### QTL analysis

A ‘forward selection’ [52] approach for identification of appropriate QTL models was implemented using the R/qtl package [53]. Single-QTL analysis using standard interval mapping and the Kruskal-Wallis rank-sum test was initially performed using the scanone function to detect genomic regions associated with downy mildew resistance across three environments. Unlike all other QTL analyses, data was not transformed prior to the Kruskal-Wallis test. LOD thresholds for significance of QTL level were determined by separate permutation tests with 1,000 iterations at α = 0.05. 1.5-LOD support intervals were calculated for all significant QTL detected.

A subsequent two-dimensional (2D) analysis was performed using the scantwo function to detect QTL pairs on separate LGs. Permutation tests were again performed with 1,000 iterations to determine significant LOD thresholds for the joint (full), conditional-interactive, interaction, additive, and conditional-additive two QTL models. An additional genome-wide scan for locus pairs within LGs was performed to account for potentially linked QTL.

Finally, a multiple-QTL model (MQM) was implemented using the fitqtl function to fit the appropriate linear model with the QTL detected from single and 2D analyses, represented as main effects. Analysis of variance (ANOVA) was performed to determine significant QTL effects and percentage of phenotypic variance explained (PVE) by each QTL. Genotype means and standard error for significant QTL were calculated and plotted using R/qtl.

## Results

### EST-SSR markers

**Fig 1:**
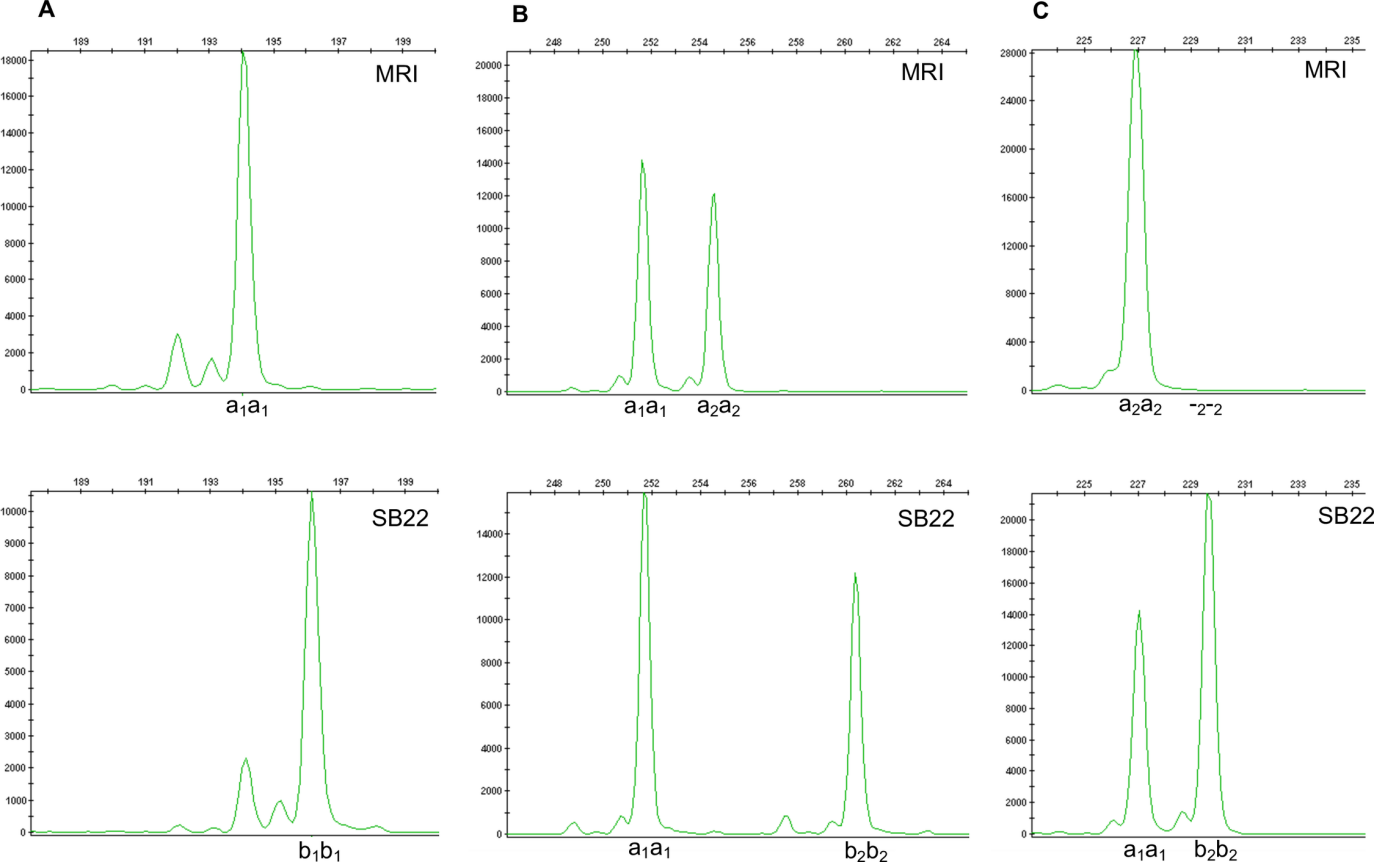
Polymorphic EST-SSR genotypes observed among subgenomes of homozygous grandparent genotypes MRI and SB22. Electropherogram plots for alleles represented as peaks with size (nucleotides) represented along the x-axis. (A) Marker OBNJR2sg34 genotype: One polymorphic locus within a single subgenome (arbitrarily designated with subscript 1) corresponding to MRI genotype a_2_a_2_ and SB22 genotype b_2_b_2_ with expected F_2_ segregation ratio 1:2:1 (a_2_a_2_:a_2_b_2_:b_2_b_2_). (B) Marker OBNJR3cn328 genotype: One monomorphic locus corresponding to a single sub-genome genotype a_1_a_1_ and one polymorphic locus in another sub-genome (arbitrarily designated with subscript 2) with expected F_2_ segregation ratio 1:2:1 (a_2_a_2_:a_2_b_2_:b_2_b_2_). (C) Marker OBNJR3cn80 genotype: Monomorphic locus a_1_a_1_ and polymorphic locus in another sub-genome corresponding to MRI null genotype -_2_−_2_ and SB22 genotype b_2_b_2_ with expected F_2_ segregation ratio 3:1 (-_2_−_2_: b_2_b_2_).

Among 240 EST-SSR markers used to genotype the grandparents, 142 primer pairs demonstrated clean (unambiguous) PCR amplification in which 1–4 unique amplicons (alleles) were classified as ‘functional’. Forty primer pairs (28.2%) (Table 1) were polymorphic and could be grouped into three bi-parental dual-homozygous genotypes: (i) one polymorphic, bi-allelic locus in one sub-genome and no locus present in the second sub-genome (Fig 1A); (ii) one monomorphic locus in one sub-genome and one polymorphic, bi-allelic locus in a second sub-genome (Fig 1B); and (iii) one monomorphic locus in one sub-genome and one locus with one allele present or absent (null) in a second sub-genome (Fig 1C). In scenarios (i) and (ii) the polymorphic locus was fit to an F_2_ segregation ratio of 1:2:1 (a_2_a_2_:a_2_b_2_:b_2_b_2_) while in scenario (iii) the polymorphic locus was fit to an F_2_ segregation ratio of 3:1 (b_2_b_2_:a_2_a_2_+a_2_b_2_).

**Table 1:** Description of 42 mapped EST-SSR markers in the MRIxSB22 linkage map indicating nucleotide sequence source, repeat motif, linkage group, centimorgan position and chi-square goodness-of-fit test results.

Marker^a^	Source^b^	Motif^c^	LG	Position (cM)	Ratio	χ2	*P*^d^
**OBNJR3cn391**	Contig3280	(TCA)5	1	16.4	3:1	3.33	<0.10
**OBNJR2cn104**	Contig3401	(CT)14	1	19.0	3:1	0.24	-
**OBNJR3sg98**	DY337354	(CCT)8	1	19.2	3:1	2.97	<0.10
**OBNJR3cn201**	Contig1783	(GAA)6	1	59.8	1:2:1	3.34	-
**OBNJR2sg34**	DY331634	(CT)11	2	32.4	1:2:1	0.77	-
**OBNJR2cn79**	Contig2573	(AT)9	2	32.7	3:1	0.52	-
**OBNJR3cn362**	Contig2969	(TGA)6	3	78.0	1:2:1	1.47	-
**OBNJR3cn56**	Contig582	(AGG)7	3	84.7	1:2:1	5.16	<0.10
**OBNJR2cn80**	Contig2575	(TA)12	4	90.3	1:2:1	1.68	-
**OBNJR2sg21.1**	DY339566	(TC)13	5	33.9	1:2:1	2.67	-
**OBNJR3sg19**	DY343509	(TCA)6	6	18.9	3:1	3.49	<0.10
**OBNJR2sg15**	DY340778	(GA)25	6	50.8	1:2:1	2.25	-
**OBNJR4sg06**	DY338242	(ACAA)5	7	139.4	3:1	1.65	-
**OBNJR2cn78**	Contig2475	(AT)10	8	56.4	1:2:1	1.88	-
**OBNJR2sg21.2**	DY339566	(TC)13	10	65.7	1:2:1	1.62	-
**OBNJR3cn389**	Contig3254	(GCA)8	11	132.0	3:1	0.86	-
**OBNJR4cn11**	Contig1679	(TCAC)4	12	144.0	3:1	0.06	-
**OBNJR2cn83**	Contig2631	(GA)18	12	144.4	1:2:1	2.65	-
**OBNJR2cn17**	Contig606	(AT)10	12	146.6	3:1	0.23	-
**OBNJR2sg04**	DY343638	(GA)17	13	40.2	1:2:1	5.37	<0.10
**OBNJR4cn16**	Contig2294	(CAAA)4	13	42.9	1:2:1	3.61	-
**OBNJR2sg119**	DY333250	(GTA)7	13	49.7	1:2:1	10.14	<0.01
**OBNJR2cn29**	Contig1138	(AC)16	13	54.6	1:2:1	2.25	-
**OBNJR2sg33**	DY333933	(AC)16	14	82.7	1:2:1	0.03	-
**OBNJR4cn15**	Contig2242	(GCCT)5	15	0.0	1:2:1	7.13	<0.05
**OBNJR3cn358**	Contig2910	(TCC)7	15	24.0	1:2:1	2.31	-
**OBNJR3cn356**	Contig2907	(AAG)9	15	26.8	1:2:1	0.48	-
**OBNJR2sg31**	DY336298	(AG)9	16	106.9	1:2:1	0.07	-
**OBNJR3cn328**	Contig2750	(AAG)6	16	107.5	1:2:1	0.76	-
**OBNJR3cn192**	Contig1724	(ACA)8	16	161.5	3:1	0.13	-
**OBNJR3cn243**	Contig2153	(GAA)6	17	0.0	3:1	0.06	-
**OBNJR3sg177**	DY322989	(TGC)5	17	2.5	3:1	4.28	<0.01
**OBNJR3cn401**	Contig3437	(CAG)7	17	6.4	3:1	0.97	-
**OBNJR3cn377**	Contig3126	(TAT)6	18	30.9	1:2:1	1.8	-
**OBNJR2cn92.2**	Contig3041	(CT)13	18	78.1	3:1	0.84	-
**OBNJR3cn54**	Contig573	(TTA)18	19	88.7	1:2:1	0.4	-
**OBNJR2cn92.1**	Contig3041	(CT)13	19	89.3	1:2:1	0.64	-
**OBNJR3cn217**	Contig1936	(ATT)6	20	32.1	3:1	0.37	-
**OBNJR4sg01**	DY343743	(TCCC)5	21	91.9	1:2:1	1.27	-
**OBNJR3cn239**	Contig2134	(TTC)8	24	58.0	1:2:1	1.2	-
**OBNJR2cn38**	Contig1352	(CA)13	25	56.0	1:2:1	1.2	-
**OBNJR2cn73**	Contig2250	(CT)24	26	56.5	1:2:1	7.13	<0.05

^a^SSR markers failing to map or found to be located at identical positions to other SSR markers are excluded. A single primer set resulting in two independently segregating loci are indicated by marker name followed by either ‘.1’ or ‘.2’

^b^SSRs are sourced from CAP3-assembled NCBI *O*. *basilicum* EST sequence database. NCBI genbank nucleotide accession is provided for SSRs located in EST sequences that could not be assembled into contigs.

^c^Repeat motif sequence and number reported refer to the original Genbank (parent) sequences

^d^Chi-square goodness-of-fit tests resulting in p < 0.10 indicate evidence for segregation distortion.

The majority of mapped EST-SSR markers grouped in scenarios (ii) or (iii) (Fig 1B and 1C) in which two loci (one monomorphic and one polymorphic) are amplified in individual sub-genomes (multi-locus markers). Six EST-SSR markers fit scenario (i) (Fig 1A) in which a single homozygous, polymorphic locus is amplified within a single sub-genome and segregated in 1:2:1 (single locus markers). Two additional scenarios were observed for markers OBNJR2sg21 and OBNJR2cn92, in which two independently segregating loci represented putative homeologs. The former generated two allele pairs (OBNJR2sg21.1 and OBNJR2sg21.2), both segregating independently with each pair fitting a 1:2:1 ratio. The latter generated one pair (OBNJR2cn92.1) fitting 1:2:1 segregation and a second pair (OBNJR2cn92.2) exhibiting amplification in a presence (MRI), absence (SB22) fashion fitting a 3:1 segregation ratio. Thus, 42 EST-SSR markers were generated from 40 primer sets (Table 1; S1 Table). Tests for goodness of fit provided evidence of segregation distortion for 10 (p<0.10) and 5 (p<0.05) EST-SSR markers depending on statistical stringency.

### SNP discovery and polymorphic loci development

In an effort to maximize *O*. *basilicum* genome complexity reduction double (*PstI-MspI*) and triple (*PstI-MspI* + *ApeKI*) digestion library preparations were compared on a subset of the F_2_ population (22 individuals). Triple digestion resulted in a mean DNA concentration of 3.4 ng/uL±1.6 considered too low for sequencing. Library preparation without *ApeKI* (double digestion) resulted in an approximate 3-fold library concentration increase (mean = 15.5ng/uL±3.63) adequate for sequencing. Thus, all libraries sequenced in this study were prepared by the double digestion method.

Illumina sequencing generated a total of 478,850,390 paired end reads with a guanine-cytosine (GC) content of 39–42%. 420,611,900 reads were retained following trimming (75bp), de-multiplexing and quality filtering. Six F_2_ individual samples had a ~10x lower retained read count relative to other samples and were excluded from subsequent analysis. The average number of reads for the remaining 94 F_2_ individuals was 4,021,000. The retained read count for the parents was ~4x the F_2_ read count mean with 16,940,960 and 16,973,580 for MRI and SB22, respectively (S1 Fig).

Alignment of retained reads from both parents resulted in a Catalog containing 195,123 Stacks, 47,842 SNPs and 25,363 polymorphic loci. 3,492 polymorphic loci having less than 20 missing individuals could be mapped back to the parent Catalog. 2,532 polymorphic loci contained exactly 1 SNP (S2 Table). Chi-square test of retained single-SNP loci revealed 565 with evidence for segregation distortion (p<0.10), or 22.3%, which were removed leaving 1,918 loci. Strict Chi-square (p <0.10) filtration of single SNP loci was employed to retain a bi-allelic set of loci [28]. A final set of 64 SNP markers produced identical genotypes and was removed, leaving a total of 1,954 polymorphic SNP and EST-SSR loci prior to linkage grouping.

### Linkage map construction

**Fig 2:**
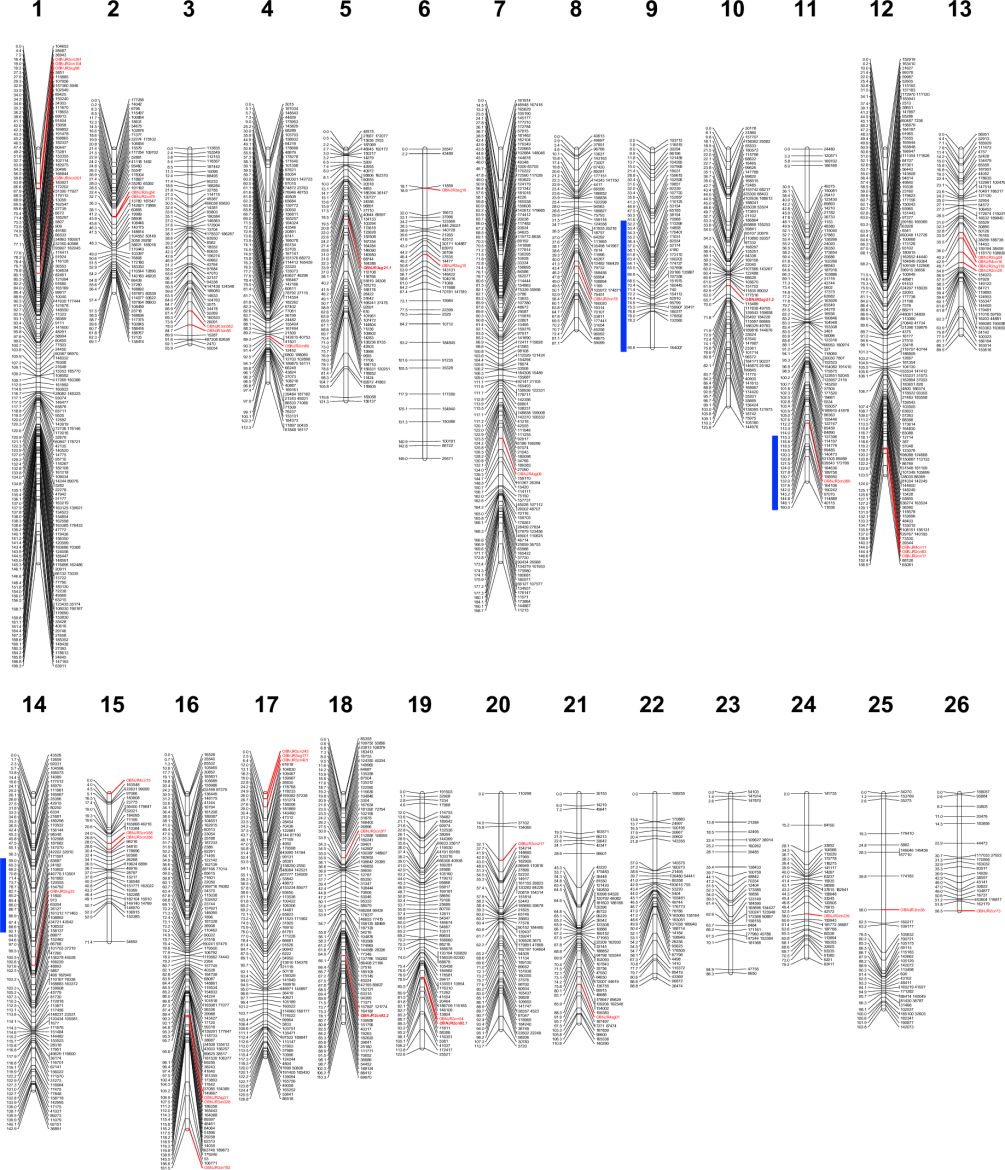
Sweet basil linkage map constructed for MRI x SB22 F_2_ intercross family. The map includes 1,847 SNP (black font) and 42 EST-SSR markers (red font) across 26 LGs for a total length of 3030.9 cM. Two pairs of multi-locus EST-SSR (bold/italic/red font) markers represent putative homeologous pairs of loci (LGs 5,10 and 18,19). Blue lines represent 1.5 LOD score confidence intervals located to the left of linkage group locations associated with downy mildew resistance.

Following removal of markers with poor support for grouping (LOD<10.0; rf<0.35) or placement, the final genetic map contained 1,847 SNP and 42 EST-SSR markers. The overall frequency of aa, ab and bb genotypes was 22.6%, 52.0% and 25.4%, respectively, reflecting an F_2_ dataset with adherence to Mendelian segregation. Grouping initially yielded 24 LGs as expected for the haploid chromosome set (n = 24). However, two LGs exhibited large and centrally located gaps of 53.5 and 50.4 cM, resulting in unusually high total map distances of 315.2 and 310.5 cM, respectively. Multiple marker placement diagnostics did not provide evidence for any poorly supported markers causing distance inflation, thus these gaps were determined real representations and warranted the division of each LG into two: LG8 (87.1 cM), LG9 (95.6 cM) and LG14 (142.0 cM), LG15 (71.4 cM). Division of these groups had no effect on marker order. The final map yielded 26 LGs (Fig 2) with an average distance of 166.6 cM and a total map distance of 3030.9 cM. SNP and EST-SSR markers were densely populated throughout the map and uniformly distributed among LGs, averaging a 1.6 cM distance between markers (Table 2).

**Table 2:** Summary of the MRIxSB22 F_2_ linkage map including number of SNPs and EST-SSRs, centimorgan length and average centimorgan distance between markers for each linkage group.

LG	SNPs	SSRs	Distance (cM)	Average distance between markers (cM)
1	158	4	199.3	1.2
2	68	2	69.1	1
3	48	2	93.9	1.9
4	90	1	112.3	1.2
5	71	1	121.3	1.7
6	37	2	149	3.9
7	141	1	198.7	1.4
8	52	1	87.1	1.7
9	45	0	95.6	2.2
10	87	1	123.8	1.4
11	85	1	160	1.9
12	143	3	156.9	1.1
13	57	4	89.5	1.5
14	97	1	142.9	1.5
15	37	3	71.4	1.8
16	105	3	161.5	1.5
17	91	3	126.8	1.4
18	91	2	110.3	1.2
19	65	2	122.6	1.9
20	60	1	113.7	1.9
21	57	1	110.6	1.9
22	40	0	88.8	2.3
23	34	0	86.3	2.6
24	31	1	79.2	2.6
25	37	1	103.8	2.8
26	20	1	56.5	2.8
Overall	1847^a^	42^a^	3030.9^a^	1.6^b^

^a^Total

^b^Average centimorgan distance

Forty-two SSR markers mapped across 23 of the 26 LGs (Table 2), providing critical PCR-based “anchor” markers for potential comparison to future sweet basil linkage maps. Two primer sets resulted in two pairs of markers (OBNJR2sg21.1, OBNJR2sg21.2 and OBNJR2cn92.1, OBNJR2cn92.2) mapping to 4 LGs and may represent two homeologous chromosome sets (LGs 5,10 and 18,19) (Fig 2).

### Downy mildew response

**Fig 3:**
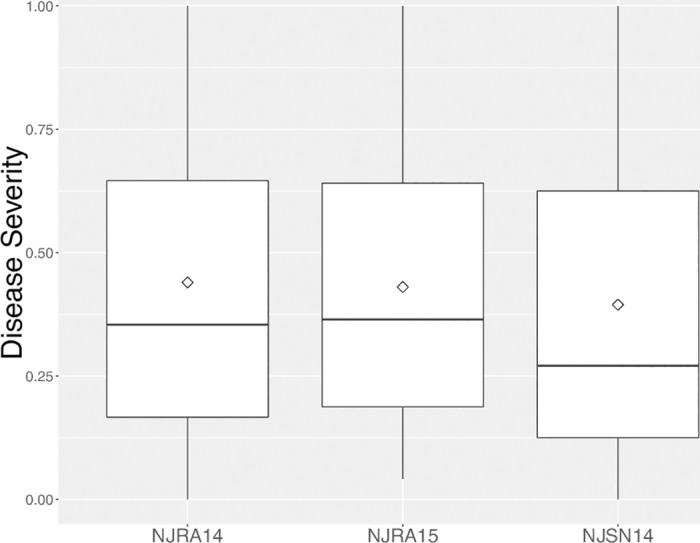
Frequency distribution of disease severity in the MRI x SB22 F_2_ mapping population across three environments. Codes for each environment are shown on the x-axis and correspond to data recorded in 2014 in southern New Jersey (NJRA14), 2015 in southern New Jersey (NJRA15) and northern New Jersey in 2014 (NJSN14). Disease severity measured on a scale in which 0 = lowest possible severity score and 1 = highest possible severity score.

Downy mildew response among individuals evaluated in 2015 was similar to previously reported results in 2014 [19]. Both grandparents were consistent in their differential response to downy mildew with SB22 >0.96 disease severity and MRI < 0.04 disease severity (Fig 3). The F_2_ population mean for NJRA15 was 0.43 as compared to 0.44 and 0.39 for NJRA14 and NJSN14, respectively. The distribution across three environments demonstrates some skewness towards a disease resistance response as previously described [19] (Fig 3). Square root transformation was applied to F_2_ phenotypic data, resulting in normally distributed residual variance across the three environments.

### Detection of QTL conferring downy mildew resistance

**Fig 4:**
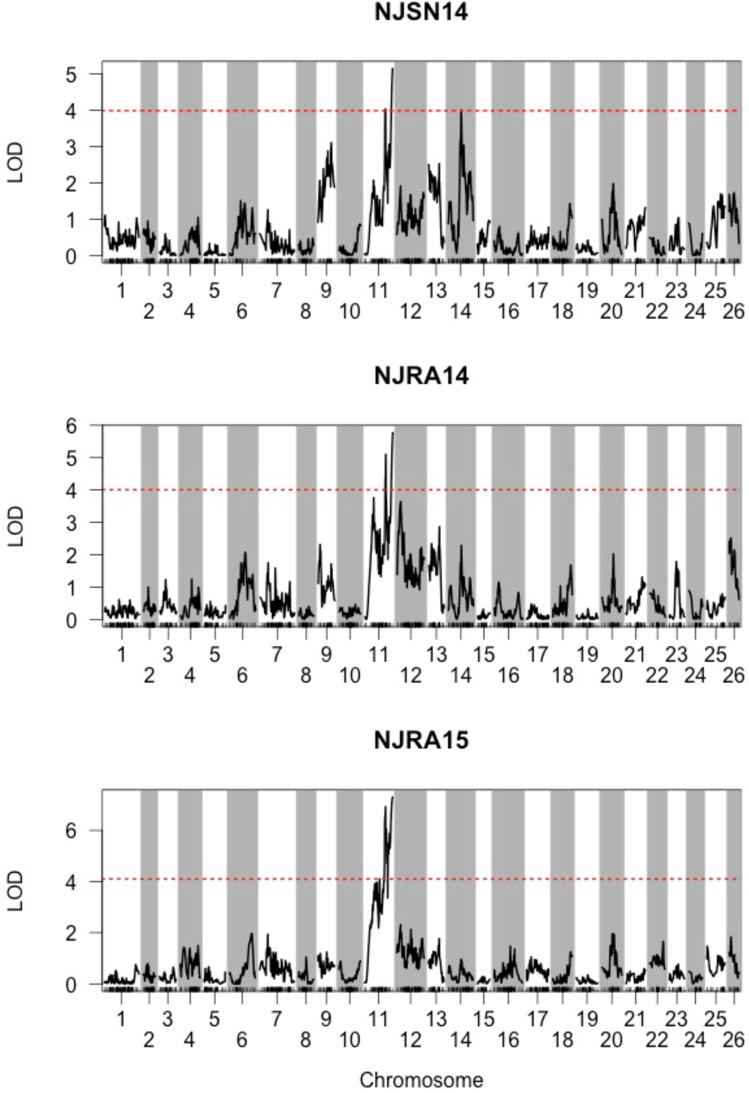
Detection of major downy mildew resistance QTL *dm11*.*1* across three environments. LOD scores for genome-wide scan using square-root transformed phenotype data from three environments: NJSN14 (northern New Jersey; 2014), NJRA14 (southern New Jersey; 2014) and NJRA15 (southern New Jersey; 2015). Significant LOD thresholds (α = 0.05) were calculated by permutation tests with 1,000 iterations and are shown with red, dashed horizontal lines.

Initial interval mapping QTL analysis detected one LG region surpassing calculated LOD thresholds (α = 0.05) 3.99, 4.01, and 4.10 corresponding to environments NJSN14, NJRA14 and NJRA15, respectively (**Fig 4**). Results of the Kruskal-Wallis test confirmed the significance of this region with LOD scores for these respective environments of 3.74, 5.08 and 5.69. A maximum LOD score was associated with the most distal end of LG 11 closest to SNP marker ‘11636’, which was renamed *dm11*.*1*. A 1.5 LOD confidence interval spanned approximately 45 cM from the location of *dm11*.*1* (**Fig 2**).

A 2D genome scan identified two additional QTL, located on LG 9 at 74.9 cM and LG 14 at 73.7 cM, renamed *dm9*.*1* and *dm14*.*1*, respectively. Interestingly, these QTL were detected (p<0.05) in environment NJSN14, but not in NJRA14 or NJRA15. The two-QTL model identified in NJSN14 provided evidence that *dm9*.*1* and *dm14*.*1* each act independently with *dm11*.*1*, thus forming two pairs: *dm9*.*1*, *dm11*.*1* and *dm11*.*1*, *dm14*.*1*. In both pairs the following two-QTL models were found to be significant: additive (LOD≥8.17; p≤0.003) and additive-conditional (LOD≥4.53; p≤0.016). These results indicate a single-QTL model is inadequate and that downy mildew resistance in at least one environment (NJSN14) should be modeled with multiple QTLs. Strong evidence (p≤0.003) was provided for the pairwise additive model suggesting that the detected locus pairs act additively to affect response to downy mildew in the MRI x SB22 F_2_ population.

Significance of two QTL pairs provided evidence for 3-QTL in at least one environment (NJSN14), necessitating the inclusion of *dm9*.*1*, *dm11*.*1* and *dm14*.*1* in a MQM. Results of the MQM analysis across all three environments indicated that *dm11*.*1* represents a ‘major QTL’ that consistently explained the greatest percentage of phenotypic variance (20.6–28.2%). The *dm14*.*1* QTL was well-supported (LOD = 3.3; p<0.001) in the NJRA14 environment with 10.5% PVE, but was not detected (p = 0.11) in single 2015 environment (NJRA15). Less support (LOD = 2.1; p<0.012) was provided for *dm9*.*1* in NJRA14 and explained 6.5% of phenotypic variance (Table 3). Similarly, in NJRA15 *dm9*.*1* was weakly detected (p = 0.047) with 5.5% PVE. LOD scores and phenotypic effect of these two QTL were more pronounced in environment NJSN14 where *dm9*.*1* (LOD = 5.8; p<0.001) and *dm14*.*1* (LOD = 6.5; p<0.001) explained 16.1% and 18.4%, respectively. Given their variable and generally lower contribution across environments *dm9*.*1* and *dm14*.*1* were considered ‘minor QTL’.

**Table 3:** Summary of three downy mildew resistance QTL detected using a multiple QTL model (MQM) across three environments.

QTL	LG	Position (cM)	SNP^a^	Confidence Interval (cM)^b^	Environment	LOD	*P*^c^	PVE (%)^d^
***dm9*.*1***	9	74.9	95799	51.9–95.6	NJSN14	5.8	<0.001	16.1
		-	-	-	NJRA14	2.1	0.012	6.5
		-	-	-	NJRA15	1.5	0.047	5.5
***dm11*.*1***	11	160.0	11636	115.3–160.0	NJSN14	7.2	<0.001	20.6
		160.0	11636	115.3–160.0	NJRA14	6.7	<0.001	23.3
		160.0	11636	114.0–160.0	NJRA15	6.5	<0.001	28.2
***dm14*.*1***	14	73.7	120555	65.3–92.1	NJSN14	6.6	<0.001	18.4
		73.7	120555	65.3–131.0	NJRA14	3.3	<0.001	10.5
		-	-	-	NJRA15	1.1	0.109	3.9

^a^Single nucleotide polymorphism (SNP) marker located in closest proximity to the QTL location

^b^1.5 LOD score intervals shown or significant (P<0.01) QTL only

^c^*P*-values represent the significance of LOD scores determined by permutation tests with 1,000 iterations at α = 0.05

^d^Percent phenotypic variance explained

**Fig 5:**
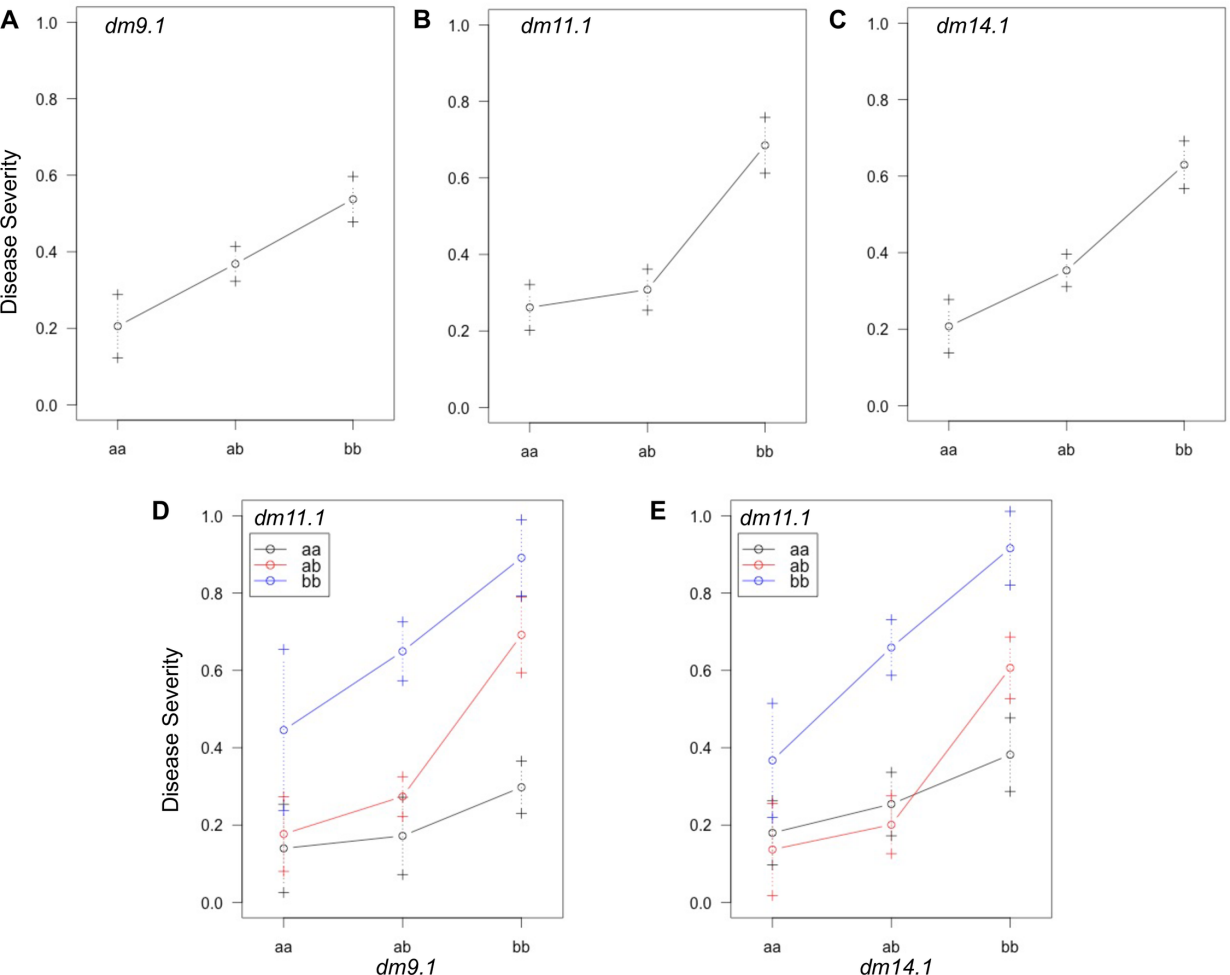
Effect and interaction plots for three QTL detected in environment NJSN14. MRI x SB22 F_2_ genotype means (circles) ± 1 SE (error bars) for (A) minor QTL *dm9*.1, (B) major QTL *dm11*.*1* and (C) minor *dm14*.*1*. Two-QTL genotype by genotype means ± 1 SE for (D) *dm11*.*1* by *dm9*.*1* and (E) *dm11*.*1* by *dm14*.*1*. Allele ‘a’ is inherited from downy mildew resistant grandparent MRI and allele ‘b’ is inherited from susceptible grandparent SB22. Error bars represent ±1 SE.

Genotypes for QTL detected in the 2D genome scan were examined for their effect on downy mildew response in environment NJSN14 (Fig 5). In each QTL, ‘a’ alleles inherited from resistant parent MRI were associated with a lower F_2_ mean downy mildew (disease) severity, while the ‘b’ alleles from susceptible parent SB22 were associated with a higher mean disease severity. Individuals with *dm11*.*1* genotypes ‘aa’ or ‘ab’ had similarly low means of 0.25±0.06 and 0.34±0.05, as compared to 0.63±0.07 for the ‘bb’ genotype (Table 4). Proximity of the ‘ab’ mean to the MRI genotype, ‘aa’ (Fig 5B), demonstrated dominant gene effects influence *dm11*.*1*-conferred downy mildew resistance. In contrast, the homozygote (‘aa’)—heterozygote (‘ab’)—homozygote (‘bb’) trend for *dm9*.*1* (Fig 5A) and *dm14*.*1* (Fig 5C) appears relatively linear suggesting additive gene effects have greater influence over the response to downy mildew.

**Table 4:** F_2_ means for downy mildew (disease) response in environment NJSN14 according to QTL genotype.

**QTL**	**aa**	**ab**	**bb**
*dm9*.*1*	0.21±0.08	0.37±0.05	0.54±0.06
*dm11*.*1*	0.25±0.06	0.34±0.05	0.66±0.07
*dm14*.*1*	0.21±0.07	0.35±0.04	0.63±0.06

The genotypes of *dm9*.*1* (Fig 5A) and *dm14*.*1* (Fig 5C) were evaluated in combination with *dm11*.*1* genotypes and demonstrated a similar effect across interacting genotype classes. In the case of *dm9*.*1* in combination with *dm11*.*1*, dual ‘aa’ homozygotes conferred the strongest resistance (0.14±0.08) while the dual ‘bb’ homozygotes result in highest mean disease severity (0.90±0.11). When *dm11*.*1* is considered with *dm14*.*1* dual ‘aa’ and ‘bb’ homozygotes result in a mean response of 0.18±0.11 and 0.97±0.11, respectively. An intermediate phenotype was previously hypothesized for the MRI x SB22 F_2_ population [19] and is supported in the presence of 3 ‘b’ alleles in both cases of QTL pairs (Fig 5D and 5E). Three ‘b’ alleles can be achieved with one heterozygous locus, one homozygous ‘bb’ locus and the reciprocal (genotypes at each locus switched). In the case of a heterozygous *dm11*.*1* and homozygous *dm14*.*1*, mean response is 0.59±0.07 compared to 0.69±0.09 for the reciprocal (Table 5). When considered with their associated standard errors, these two genotype scenarios appear to be associated with an intermediate response (Fig 5E). Results are similar for *dm11*.*1* and *dm14*.*1*, where the range is 0.65±0.08–0.66±0.11 (Table 5). Investigation of segregating alleles at multiple loci demonstrates that while *dm11*.*1* is most influential, it is not acting independently and a more complete informative model must include additive minor effect QTL.

**Table 5:** F_2_ means for downy mildew (disease) response in environment NJSN14 according to two-QTL genotype by genotype combinations.

	*dm9*.*1*			*dm14*.*1*		
*dm11*.*1*	aa	ab	bb	aa	ab	bb
aa	0.14±0.12	0.19±0.11	0.30±0.07	0.18±0.08	0.20±0.08	0.38±0.10
ab	0.18±0.10	0.27±0.05	0.67±0.11	0.16±0.11	0.22±0.07	0.60±0.07
bb	0.35±0.15	0.654±0.08	0.90±0.11	0.30±0.11	0.69±0.09	0.98±0.11

Both *dm9*.*1* and *dm14*.*1* separately fit a two-QTL additive model with *dm11*.*1*. Means for each genotype by genotype effect colored on a scale from green (low disease severity) to yellow (intermediate disease severity) to red (high disease severity).

## Discussion

### EST-SSR and SNP genotyping

Until recently, linkage mapping of non-model species relied heavily upon PCR-based markers such as SSRs and AFLPs, providing valuable but costly genotype data that were often inadequate for achieving dense genome coverage. Reduced representation sequencing has substantially decreased the economic and bioinformatics hurdles required to genotype and map plant species lacking genomic resources [54–56]. Little to no genomic sequence data have been made available for basil with the exception of a recent *Ocimum sanctum* draft genome assembly estimated to be 386 Mbp [57]. This massive disparity in *O*. *sanctum* genome size relative to *O*. *basilicum* suggests massive accumulation of genomic content and genetic divergence likely to exceed a threshold (1–5%) [58] at which mapping short reads would be successful. The *O*. *sanctum* assembly is therefore unlikely to provide utility for read alignment or validation of physical marker positions, leaving *O*. *basilicum* currently without a reference genome.

Implementation of *PstI-MspI* ddRADseq [47] facilitated high-throughput *de novo* SNP discovery and made feasible the construction of a first generation sweet basil linkage map. The Stacks *de novo* genotyping software pipeline performed effectively with ddRADseq sequence data, generating over 25,000 polymorphic loci between grandparents without a reference genome. Strict filtration for single-SNP markers from dual-homozygous grandparents absent of F_2_ segregation distortion (p<0.10) resulted in a greater than 10-fold reduction in the final number of mappable loci. However, 1,887 bi-allelic SNP markers were identified, successfully generating a dataset imputable as a diploid intercross.

The 22.3% rate of segregation distortion (p<0.10) observed for the MRIxSB22 F_2_ population is comparable to that of better characterized allopolyploids such as strawberry (22.4%. p < 0.05) [59], wheat (34%) [60] and peanut (39.1%, P<0.05) [61]. Deviation from expected segregation ratios can be attributed to various reproductive biological factors [62,63] and are typically higher in mapping populations derived from interspecific crosses [64,65]. F_1_ progeny obtained from hybridization of MRI and SB22 exhibited no sterility suggesting both grandparents belong to the same species, *O*. *basilicum*. Furthermore, preferential pairing is demonstrated by the frequent occurrence of predictable disomic inheritance patterns of EST-SSR loci (1:2:1 and 3:1), indicating divergent subgenomes that are less likely to exhibit multivalent chromosome behavior during meiosis. Instead, SNP markers failing to fit expected genotype class segregation patterns may be due to mistaken merging of sequence reads from homeologs subsequently called as homologous polymorphic loci within the same subgenome [22,27]. Availability of known diploid ancestors for the *Gossypium* sp. A_T_ and D_T_ subgenomes facilitated identification of putative SNP locus homologs [31]. In the absence of such resources, precautionary removal of loci with poor chi square goodness of fit to a 1:2:1 ratio was necessary to avoid inclusion of potential false-positive loci.

Although not critical to saturation of the linkage map, development and mapping of 42 EST-SSRs (28.1% of functional markers) (Table 1) provided needed evidence for disomic inheritance. A similar approach was recently employed to determined disomic inheritance of SSR markers in S_1_ populations of *Cynodon dactylon* [66]. Eighty-seven of the 142 (61.3%) functional EST-SSR markers amplified two or more alleles, which is comparable to the 66.2% reported in a comparison inbred *Brassica* species [29]. In an inbred allotetraploid the maximum number of alleles represented by a single SSR marker in one genotype should not exceed two (Fig 1). Occurrence of 3 or 4 alleles per locus in a single grandparent (7.3% in SB22 and 8.7% in MRI) suggests a small percentage of heterozygous loci in one (3 alleles) or both (4 alleles) subgenomes. Although these SSR markers were not mappable, observation of tri- and tetra-allelic loci provide further supporting evidence for an allotetraploid genome structure. In the absence of knowledge concerning *O*. *basilicum* genome structure, initial EST-SSR genotype information provided needed evidence for disomic inheritance that could be fit to a traditional diploid intercross model for further investigation.

### A first generation sweet basil linkage map

This study resulted in a sweet basil linkage map with 1,847 SNP and 42 SSR markers covering 3030.9 cM. The 26 LGs reported include two LG sets (8,9 and 14,15) that were originally merged and >300 cM in length. Observation of ~50 cM gaps in these two LGs and evidence of weak linkages among markers on either side of each gap informed the decision to divide these two LGs. This conservative approach avoided the possibility of mistakenly combining separate chromosomes and generated 26 highly supported LGs with evenly distributed markers (Table 2) and no major gaps (Fig 3). A similar result was recently reported for cultivated strawberry from a ddRADseq-based linkage mapping generating 31 LGs, three greater than the expected 28 for the known haploid chromosome set (n = 28) [56].

EST-SSR markers were successfully distributed across 23 of the 26 linkage groups in this study (Fig 1). SSR markers derived from genic sequence databases such as EST libraries are more likely to be transferrable across diverse germplasm and are thus ideal ‘anchor’ markers for comparison of linkage maps across populations [29,67]. Clustering of 2–4 EST-SSR markers was common on the MRI x SB22 linkage map. Among 7 LGs, SSRs (between 2 and 3) mapped within very short intervals (2.3 ± 1.7 cM) (Fig 2). The *O*. *basilicum* NCBI EST library is based largely on tissue-specific cDNA sequences from transcripts related to synthesis of secondary metabolites [68]. The occurrence of the EST-SSR groupings suggest they may be derived from transcripts contributing to a given biosynthetic pathway, potentially clustered in a single genomic region. Interestingly, two multi-locus SSRs (OBNJR2sg21 and OBNJR2cn92) mapped to 4 unique LGs with no evidence for segregation distortion (p>0.10) (Table 1). These four LGs potentially represent two homeologous chromosome sets (LGs 5,10 and 18,19), however, further investigation is needed to build support for this hypothesis.

### Downy mildew resistance QTL detection

One major QTL, *dm11*.*1*, and two minor QTL, *dm9*.*1* and *dm14*.*1*, were associated with response to downy mildew in the MRI x SB22 F_2_ mapping population. Major QTL *dm11*.*1* was located on the most distal end of LG 11 (160.0 cM), close to SNP ‘11636’ and explained 20.6–28.2% of phenotypic variance across three environments. The contribution of minor QTL *dm9*.*1* and *dm14*.*1* was lower in NJRA14 and NJRA15 where the combined PVE was 17.0% and 9.4%, respectively. The 2014 F_2_ mean disease severity in southern NJ (NJRA14) was significantly higher (p<0.05) than northern NJ (NJSN14) [19]. Despite this difference in disease pressure all three QTL were detected (p<0.05) in both 2014 NJ locations. However, the PVE in NJRA14 by minor QTL decreased substantially relative to NJSN14, while PVE by *dm11*.*1* was slightly increased by 3.3% (Table 3). Only *dm9*.*1* and *dm11*.*1* were detected in the 2015 environment NJRA15, while *dm14*.*1* could not be identified (p = 0.109). PVE of 28.2% for *dm11*.*1* was highest in this environment, while that of *dm9*.*1* was comparable to NJRA14 (Table 3). Higher F_2_ mean disease severities of 0.44 and 0.43 for the southern NJ location in 2014 and 2015, respectively, suggest an interaction of disease pressure and/or location with QTL effect. However, it should be noted that the population size was reduced to 80 individuals in the NJRA15, which may have affected QTL resolution in this environment. PVE of *dm11*.*1* was negatively correlated with *dm9*.*1* and *dm14*.*1* across all environments (Table 3), suggesting that the effect of minor and major QTL may be inversely related when subject to different environmental conditions (eg. disease severity).

When considered in isolation, this *dm11*.*1* would appear to act as a single-dominant gene in which one ‘a’ allele is sufficient to confer a resistant downy mildew response (≤0.34±0.05) (Table 5) (Fig 5B) as has been observed in *Brassica* spp. [69], spinach [39] grape [41]. However, the single, dominant gene hypothesis (0–0.33 = resistant individual and 0.34 ≤ susceptible individual) was previously rejected by Pyne et al. [19] (Chi-squared test, p <0.01) in F_2_ and backcross populations using phenotypic data from NJSN14 and NJRA14, concluding that at least one additional locus was affecting downy mildew response. In this study, detection of additional *dm9*.*1* and *dm14*.*1* in additive-two and multiple QTL models support the previous phenotypic-based findings.

Greatest support for QTL *dm9*.*1* and *dm14*.*1* (LOD> 5.8; p<0.001) occurred in environment NJSN14 (northern New Jersey; 2014) where these QTL were associated with 34.5% of PVE (Table 3). Consideration of genotype effects for both QTL in this environment demonstrates a severe consequence (high susceptibility) for individuals with dual homozygous ‘b’ alleles (Fig 5D and 5E) in which susceptibility is additive as F_2_ mean disease severity surpasses the maximum (0.66±0.07) observed for any individual ‘bb’ QTL genotype (Fig 5D and 5E). This result is supported by previously identified highly significant (p<0.001) positive additive (‘bb’) x additive (‘bb’) effects from a joint scaling test using the MRI x SB22 full-sibling family indicating the presence of homozygous loci with an increase in susceptibility [19]. Successive subtraction of SB22 ‘b’ alleles from either of the *dm11*.*1*-*dm9*.*1* or *dm11*.*1*-*dm14*.*1* QTL pairs detected in 2D QTL analysis results in an incremental reduction of F_2_ mean disease severity (i.e., increased resistance). This two-QTL system therefore provides evidence for at least three downy mildew response classes: susceptible (4 ‘b’ alleles), intermediate (3 ‘b’ alleles) and resistant (0–2 ‘b’ alleles) (Fig 5D and 5E).

Dominant gene action conferred by the ‘a’ allele in *dm11*.*1* appears to be capable of countering the susceptibility effect of ‘b’ alleles in either minor QTL when 1–2 ‘b’ alleles are present. Resistance begins to break down, however, with the accumulation 3 or 4 ‘b’ alleles in either QTL (Fig 5D and 5E). Again, these results are supported by a previously described positive additive (‘bb’) x dominant (‘ab’) effect [19] through resistance of the heterozygous *dm11*.*1* locus being reduced by homozygous ‘bb’ loci in either minor QTL. The resistance-reducing effect of a ‘bb’ genotype in *dm11*.*1* with an ‘aa’ genotype in *dm9*.*1* was greater than the reciprocal (‘aa’ genotype in *dm11*.*1* with ‘bb’ *dm9*.*1*) (Fig 5D). In contrast, comparison of F_2_ means for reciprocal, opposing homozygous genotype combination in *dm11*.*1* and *dm14*.*1* resulted in similar mean disease severity (Fig 5E). In both cases, a relatively high SE (0.07–0.10) demonstrates variability in downy mildew response when the recessive ‘bb’ *dm11*.*1* genotype is present with the ‘aa’ genotype of either minor QTL, resulting in some loss of resistance response within the population. Chi-square goodness of fit to complementary and recessive epistatic gene models in F_2_ and backcross generations from phenotypic data suggested dominant effects were needed to confer resistance. A dominant (‘ab’) x dominant (‘ab’) gene effect was thus expected but unsupported (p = 0.769) [19]. QTL in this study provide evidence for a more complex gene model with a major, single dominant and two minor, additive QTL (Table 3).

It is clear that applied resistance breeding would benefit from ensuring germplasm have the *dm11*.*1* ‘aa’ (MRI) genotype as a heterozygous locus at *dm11*.*1* will result in loss of resistance through segregation during self-pollinated seed propagation. Given the potential increased susceptibility effect of the ‘b’ allele, its removal from each QTL (‘aa’ genotype) would be preferable to ensure a high level of stable resistance. A similar model was identified in the GR x Ice RIL population in which the homozygous Iceberg genotype at two QTL conferred significantly higher resistance to downy mildew [70].

Quantitative and qualitative forms of downy mildew resistance have been reported in multiple plant species such as *Cucumis* spp. where sources of host resistance have been identified and characterized for decades [71]. Quantitative resistance, while less susceptible to breakdown, is subject to greater variation [71] across environments as was observed for the QTL *dm9*.*1* and *dm14*.*1* detected in this study (9.4–34.5% PVE). A similar range of PVE (15–30%) for downy mildew response was reported for two additive QTL in cucumber F_2:3_ families across three environments [40]. Qualitative downy mildew resistance, while more prone to breakdown, is less vulnerable to environmental interaction and often associated with gene-for-gene host-pathogen interaction. Major QTL *dm11*.*1* was detected in a distal region of LG 11 with resistance conferred from grandparent MRI by the dominant ‘a’ allele. The downy mildew resistance dominant locus *Rpv3* was also detected in a distal chromosomal region of *V*. *vinifera* ‘Bianca’ known to contain NBS-LRR gene clusters [41]. Development of a *de novo* metatranscriptomics pipeline [72] for *O*. *basilicum* provides a platform for identification of resistant gene motifs, which could potentially be mapped to genomic regions containing QTL such as *dm11*.*1* for functional characterization.

RADseq approaches have changed the landscape of linkage and QTL mapping for non-model plant species by introducing low-cost, high-throughput, *de novo* SNP discovery. The inexpensive acquisition of tens or hundreds of thousands of SNP markers allow researchers working with poorly understood genomes to be ‘picky’, applying strict filtration to retain >1,000 high-quality SNPs with predictable segregation patterns. This reduces the burden to generate large numbers of labor-intensive, costly markers such as SSRs, instead utilizing a smaller subset to serve as ‘anchor’ markers for subsequent map comparison. This genotyping approach high-SNP/low-SSR genotyping approach has facilitated map construction in peach [55], strawberry [56], sesame [73] and lentil [74]. In this study, the power of this approach is further demonstrated through the development of a sweet basil linkage map and QTL detection.

## Conclusions

Genetic study of non-model, horticultural species such as sweet basil are often neglected due to perceived low economic importance; however, this renders such crops vulnerable to rapid and wide-spread decline upon introduction of new plant pathogens. *P*. *belbahrii* now causes worldwide economic losses with no available resistant sweet basil cultivars. In this study, a set of EST-SSR markers were developed and mapped in the MRI x SB22 F_2_ sweet basil mapping population providing molecular evidence of disomic inheritance. Effective filtration of ddRADseq SNP markers generated 1,847 bi-allelic, polymorphic markers in the absence of a reference genome. This novel genotyping approach facilitated construction of the first linkage map for sweet basil. The utility of this map was demonstrated through identification of one major and two minor QTL associated with downy mildew resistance, largely supporting a previous report using phenotypic data only. Results provide the first steps towards the development of molecular tools for accelerated sweet basil breeding strategies.

## Supporting information

S1 Table Mapped EST-SSR primer sequences. (XLSX)

S2 Table Single-SNP sequences used to construct genetic map. (XLSX)

S1 Fig Stacks denovomap.pl output. Distribution (bar graph) of Stacks, SNPs and Polymorphic Loci identified in the MRIxSB22 F2 population.
(PDF)
