# Supplementary figures and images for: A first linkage map and downy mildew resistance QTL discovery for sweet basil (Ocimum basilicum) facilitated by double digestion restriction site associated DNA sequencing (ddRADseq)
Source: PLoS One. 2017 Sep 18;12(9):e0184319. doi: 10.1371/journal.pone.0184319 (PMC5603166; doi:10.1371/journal.pone.0184319)

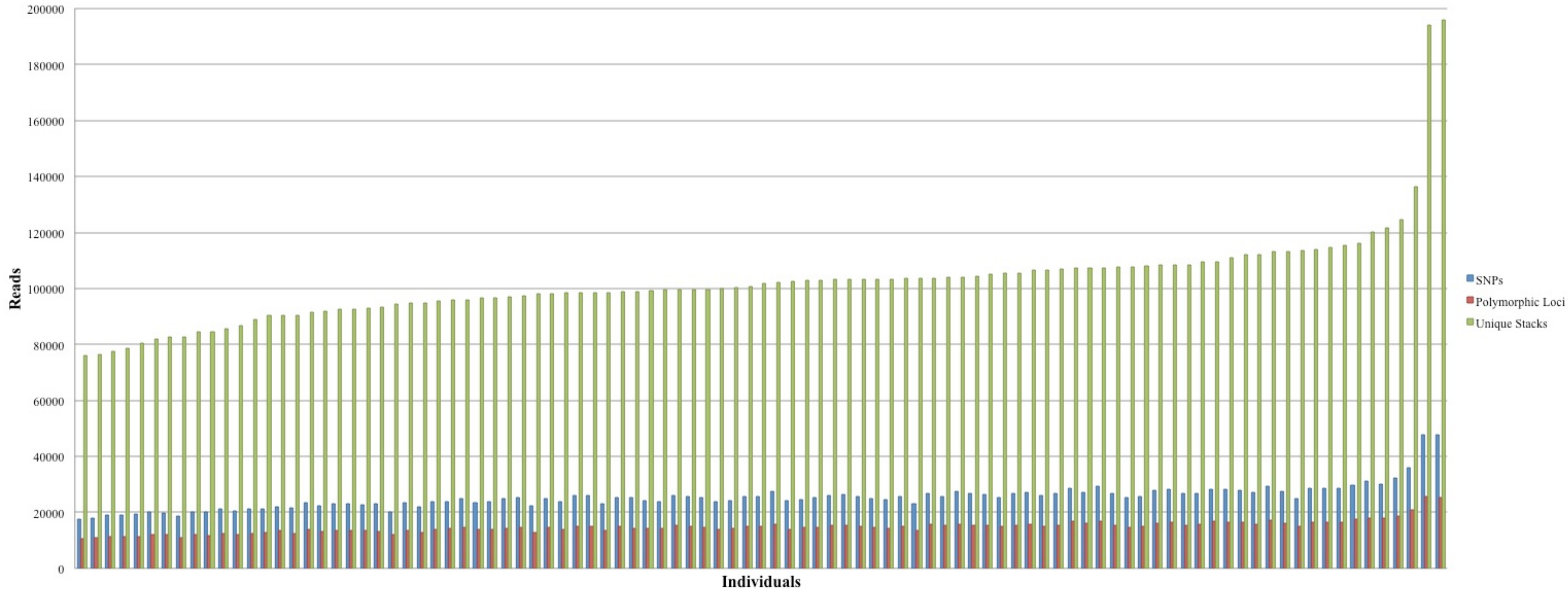

Supplement: S1 Fig — Distribution (bar graph) of Stacks, SNPs and Polymorphic Loci identified in the MRIxSB22 F2 population. (PDF) [file pone.0184319.s003.pdf]
